# Dr. Nelson Waltow Wilson

**Published:** 1915-10

**Authors:** 


### OBITUARY

**Readers are requested to report promptly the death of all physicians in Western New York, or former residents of this region, or graduates of any medical school in Western New York, and to notify the families of the deceased of our desire to publish adequate obituary notices.**

**Dr. Nelson Waltow Wilson**, Buffalo 1898, the well known genito-urinary surgeon of Buffalo, died suddenly in a New York theatre, August 30, while on a vacation. He was born July 30, 1865, in New York City. His early manhood was devoted to newspaper work, on the N. Y. Times, World and Sun. In 1890, he came to Buffalo as City Editor of the News. He married Miss Charlotte Inman, February 17, 1892. During the Spanish War, he acted as Post Surgeon at Ft. Porter and was Assistant Surgeon at the Pan-American Exposition and one of those who attended the late President McKinley. He was for several years, one of the editorial staff of this journal. He was on the staff of the Buffalo General Hospital and was Instructor in G. U. Diseases in the University of Buffalo. He was a Fellow of the American College of Surgeons, First Lieutenant in the U. S. Medical Reserve Corps. He was a 32d degree Mason and a member of Ancient Landmarks Lodge and held membership in the Buffalo and Park Clubs.

